# Case Series of Primary Esophageal Tuberculosis

**DOI:** 10.4269/ajtmh.26-0086

**Published:** 2026-04-28

**Authors:** Hong-Mei Chen, Sha-Na Zhang, Jing Tong, Chen-Guang Li, Meng-Qiu Gao, Qiang Li

**Affiliations:** ^1^ Department of Tuberculosis, Beijing Chest Hospital, Capital Medical University/Beijing Tuberculosis and Thoracic Tumor Research Institute, Beijing, China;; ^2^ Molecular Biology Laboratory, Beijing Electric Power Hospital of State Grid Corporation of China/Capital Medical University Electric Power Teaching Hospital, Beijing, China;; ^3^ The Digestive Endoscopy Center, Beijing Chest Hospital, Capital Medical University/Beijing Tuberculosis and Thoracic Tumor Research Institute, Beijing, China

## Abstract

Gastrointestinal tuberculosis is a relatively common form of extrapulmonary tuberculosis. Primary esophageal tuberculosis is rare and frequently misdiagnosed or delayed in diagnosis by clinicians. Four cases of primary esophageal tuberculosis are reported. All patients involved underwent pathological examination of their esophageal lesions, which revealed granulomas. Additionally, molecular detection confirmed the presence of *Mycobacterium tuberculosis* DNA in two cases. All patients received antituberculosis therapy and achieved good outcomes.

## INTRODUCTION

In 2019, of the 7.1 million incident tuberculosis (TB) cases notified globally, 16% involved extrapulmonary TB.^1^ In the United States, gastrointestinal TB accounts for 2.5% of extrapulmonary TB cases.^2^ Esophageal TB (ETB) is a rare extrapulmonary TB presentation that constitutes 2.8% of gastrointestinal TB cases^3^ and 0.15% of TB deaths.^4^ Meanwhile, because of the lack of specific clinical manifestations and signs of ETB, 75% of patients experienced delays in diagnosis and management.^5^ Because the clinical presentation, radiology, and gastrointestinal endoscopy findings of ETB are similar to those of esophageal cancer, it is easily misdiagnosed, with one-fifth of ETB patients being diagnosed after surgical resection.^5^^,^^6^ Because the lower esophageal sphincter prevents reflux and the esophageal peristalsis inhibits the retention of *Mycobacterium tuberculosis* (MTB) in the esophagus,^5^ primary ETB is rare, posing a significant challenge to diagnosis.

## MATERIALS AND METHODS

A search of the electronic medical record system of Beijing Chest Hospital, Capital Medical University, revealed ∼33,000 inpatient cases of TB between January 2016 and December 2025. Detailed medical records were reviewed, and four patients were definitively diagnosed with primary ETB during this period. A retrospective analysis of these patients’ demographic data, medical histories, clinical manifestations, interferon-gamma release assays (IGRAs), HIV, gastrointestinal endoscopies, esophageal lesion pathologies, computed tomography (CT), ultrasounds, anti-TB regimens, and prognoses was conducted (Table 1).

**Table 1:** Characteristics of primary esophageal tuberculosis

Variables	Case 1	Case 2	Case 3	Case 4
Age (years)	35	74	69	49
Sex	Female	Male	Male	Female
Symptoms	Dysphagia	Back pain	Dysphagia, chest pain, weight loss, and fatigue	Dysphagia, weight loss, and fatigue
Past medical history	None	None	None	None
HIV	Negative	Negative	Negative	Negative
Blood IGRA	Positive	Negative	Positive	Positive
Chest CT	Normal	A ground-glass opacity in the left upper lobe and slight thickening of the esophageal wall	Localized thickening of the esophageal wall at the level of the tracheal carina	Esophageal dilatation
Ultrasound*	Normal	Normal	Normal	Normal
Misdiagnosis	Esophageal leiomyoma	Reflux esophagitis	Esophageal cancer	–
Delayed diagnosis (months)	1	2	5	12
Gastroscopic findings before treatment	Submucosal elevation and ulceration	Ulceration	Ulceration and esophago-mediastinal fistula	Longitudinal hyperplasia of the mucosa, with localized nodules and a near-circular elevation
Distance from the incisors	25–30 cm	35–36 cm	27–29 cm	27–31 cm
Lesion size^†^	2.0 cm × 1.5 cm	1.0 cm × 1.0 cm	2.0 cm × 1.0 cm	–
EUS^‡^	A 10.68 mm × 4.56 mm spindle-shaped, well-circumscribed, homogeneous, and hypoechoic to isoechoic lesion arising from the muscularis mucosae	–	–	A 6.72 mm × 5.90 mm near-circular, heterogeneous, and hypoechoic lesion outside the esophageal wall
Pathological findings	Granulomas with necrosis and positive MTB-DNA results	Granulomas with necrosis and positive MTB-DNA results	Granulomatous inflammation with negative MTB-DNA, AFB, and PAS stain results	Granulomatous inflammation with negative MTB-DNA, AFB, and PAS stain results
Anti-TB regimen	2HREZ/10HR	2HP_2_ELfx/10HP_2_	2HREZ/10HELfx	2HREZ/10HR
Symptoms at treatment discontinuation	Relieved	Resolved	Relieved	Relieved
Gastroscopic findings at treatment discontinuation^§^	Scar-like changes with localized hyperplastic nodules	Normal	–	Scar-like changes with localized hyperplastic nodules

AFB = acid-fast bacilli; CT = computed tomography; E = ethambutol; EUS = endoscopic ultrasonography; H = isoniazid; IGRA = interferon-gamma release assays; Lfx = levofloxacin; MTB = *Mycobacterium tuberculosis*; P = rifapentine; PAS = periodic acid-Schiff; R = rifampicin; TB = tuberculosis; Z = pyrazinamide.

* Ultrasound examination sites included the abdomen and superficial lymph nodes (neck, axillary, and groin).

^†^ The lesion size of Case 4 was unknown.

^‡^ Endoscopic ultrasonography was not performed in Cases 2 and 3.

^§^ A gastroscopy was not performed in Case 3 at treatment discontinuation.

## CASE REPORTS

### Case 1.

**Figure 1.**
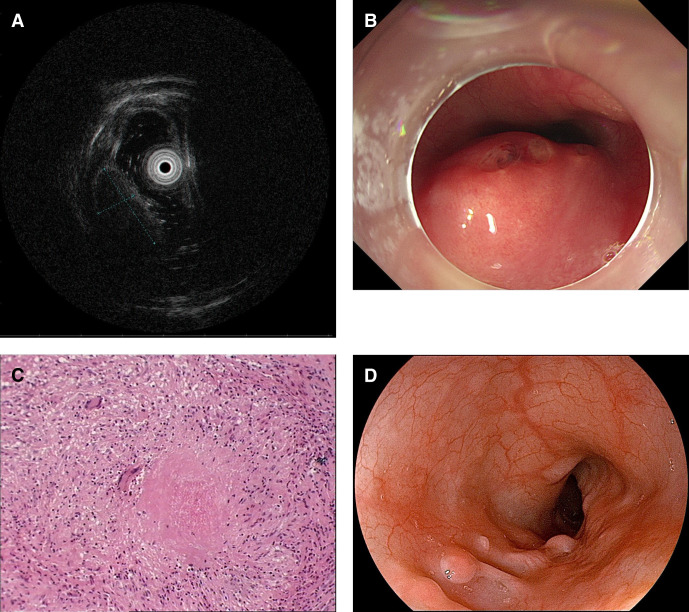
(**A**) Endoscopic ultrasonography showing a spindle-shaped, well-circumscribed, homogeneous, and hypoechoic to isoechoic lesion arising from the muscularis mucosae of the upper-middle esophagus. (**B**) Gastroscopy before treatment showing submucosal elevation in the esophagus, with scattered shallow ulcers visible on the surface. (**C**) Pathological examination showing severe acute and chronic inflammation of the esophageal mucosa and granulomatous lesions with few necroses. (**D**) Gastroscopy at the treatment discontinuation showing scar-like changes with localized hyperplastic nodules.

The patient, a 35-year-old woman, presented with dysphagia. No abnormalities were found on oral examination, and a chest CT scan revealed no abnormal findings. A gastroscopy revealed a submucosal elevation in the upper-middle esophagus. Endoscopic ultrasonography (EUS) revealed a spindle-shaped, homogeneous, and hypoechoic to isoechoic lesion in the mucosal elevation (Figure 1A). Esophageal leiomyoma was suspected by clinicians. The patient underwent a repeat gastroscopy, which revealed an ulcer in the upper-middle esophagus (Figure 1B). An endoscopic submucosal dissection was performed, and the entire mass was removed for biopsy, which revealed granulomas with necrosis (Figure 1C). Formalin-fixed paraffin-embedded tissue specimens tested positive for MTB-DNA via real-time quantitative polymerase chain reaction (RT-qPCR). Ultimately, the patient was diagnosed with primary ETB. An anti-TB regimen, which included isoniazid (H) at a dose of 300 mg, rifampicin (R) at a dose of 600 mg, ethambutol (E) at a dose of 1,000 mg, and pyrazinamide (Z) at a dose of 1,500 mg daily, was administered for 2 months, with continued H and R administration. After 12 months of treatment, a gastroscopy revealed scar-like changes accompanied by localized hyperplastic nodules (Figure 1D).

### Case 2.

The patient, a 74-year-old man, presented with back pain. His electrocardiogram and myocardial enzymes were normal. Reflux esophagitis was initially suspected. A chest CT scan revealed a ground-glass opacity in the left upper lobe and slight thickening of the esophageal wall. An abdominal CT scan revealed no abnormalities. Induced sputum smear, culture, and polymerase chain reaction (PCR) test results were all negative. Additionally, a gastroscopy revealed a mucosal ulceration in the lower esophagus. On the basis of an esophageal histology, ETB was ultimately confirmed. A regimen of H at a dose of 300 mg, E at a dose of 750 mg, levofloxacin (Lfx) at a dose of 600 mg per day, and rifapentine (P) at a dose of 450 mg twice per week was administered for 2 months, with H and P continued for 10 months. A gastroscopy revealed normal esophageal mucosa at the end of treatment. The ground-glass opacity was surgically removed, and lung cancer was diagnosed.

### Case 3.

A 69-year-old man presented with dysphagia, chest pain, weight loss, and fatigue. His heart-related test results were normal. A gastroscopy revealed an ulcerative lesion in the mid-esophagus. A follow-up gastroscopy was performed at the local hospital, revealing a 1.0 cm × 2.5 cm longitudinal fistula in the esophagus with purulent discharge but no air bubbles. Strong suspicion of esophageal cancer and esophago-mediastinal fistula was raised. On histopathological examination, granulomatous inflammation was discovered. This indicated that ETB should be considered primarily. The patient received H at a dose of 300 mg, R at a dose of 450 mg, E at a dose of 750 mg, and Z at a dose of 1,500 mg daily for 2 months. Additionally, abnormal liver function was found, with an alanine aminotransferase level of 185 U/L and an aspartate aminotransferase level of 200 U/L. The medication was adjusted to H, E, and Lfx at doses of 600 mg per day for 10 months. The patient’s symptoms improved, and a repeat gastroscopy performed after 6 months of treatment revealed slight esophageal mucosa roughness and depression, along with closure of the esophago-mediastinal fistula.

### Case 4.

A 49-year-old woman presented with dysphagia, weight loss, and fatigue. Three gastroscopy examinations performed at the local hospital revealed esophageal masses and ulcers in the mid- to lower esophagus, but no definitive diagnosis was made. An abdominal CT scan yielded normal results. A repeat gastroscopy revealed longitudinal hyperplasia of the mucosa, with nodules and a near-circular elevation. Histology revealed granulomatous inflammation, and ETB was ultimately considered. A regimen including H at a dose of 300 mg, R at a dose of 450 mg, E at a dose of 750 mg, and Z at a dose of 1,500 mg daily was administered for 2 months. Subsequently, the patient continued treatment with H and R for 10 months. Scar-like changes with localized hyperplastic nodules were revealed on a gastroscopy at the end of the treatment.

## DISCUSSION

Esophageal TB is rare even in countries where TB is endemic, such as China. There are two types of ETB: primary and secondary ETB. The former only involves the esophagus without evidence of TB in any other system (as in these cases), and the latter is caused by an invasion of TB lesions in other organs and is more common than primary ETB.^5^^,^^6^ It was reported in some of the literature that primary ETB constitutes less than 0.2% of all TB cases^7^; however, the present study indicates that primary ETB accounted for ∼0.012% of all TB cases during the same period. Secondary MTB infection of the esophagus occurs via multiple routes, with the most common being dissemination from TB lesions in adjacent tissues, such as the lungs, mediastinum, hilar lymph nodes, and spine.^5^ Other routes include the lymphatic and hematogenous routes.^5^ Dysphagia and chest pain are the primary symptoms of ETB; however, these symptoms often overlap with other esophageal diseases (e.g., esophageal cancer).^6^^,^^8^ Studies have revealed that the proportion of ETB among patients with dysphagia is only 0.3–0.55%,^4^^,^^9^ to the point that ETB was not considered in many studies.^5^ Tuberculosis symptoms are common in ETB^5^; however, only Cases 3 and 4 exhibited weight loss and fatigue in the present study, which may have made clinicians less vigilant about ETB. The average time to diagnosis ETB for these patients was 5 months, and Case 3 developed a serious complication in the form of an esophago-mediastinal fistula.

Chest CT scans can reveal the location of lesions, but they are nonspecific. The diagnosis of ETB usually requires the presence of TB lesions in the lungs, hilar, and mediastinal lymph nodes.^5^ Gastroscopy not only directly visualizes esophageal lesions but also helps obtain tissue for pathological examination. The mid-esophagus is the most common site of ETB, and ulcerations are most frequently observed there,^10^ as seen in these cases. Pathological examination is an important means of diagnosing ETB. However, the sensitivity of endoscopic biopsy is not high, ranging from 25% to 60.8%.^11^ For suspected ETB patients, obtaining biopsy specimens of the submucosa may improve sensitivity,^5^ as observed in Case 1. At the same time, EUS-guided fine-needle aspiration can be used to obtain biopsy specimens from submucosal lesions and adjacent lymph nodes, which is also helpful in diagnosing ETB.^5^ A review revealed that most pathological diagnoses of ETB were based on granulomatous inflammation, whereas there are few reports of using GeneXpert MTB/RIF (Cepheid, Sunnyvale, CA) and PCR.^10^ Two cases reported in the present study were confirmed by the presence of MTB-DNA detected via RT-qPCR testing; however, the levels of MTB-DNA were below the drug resistance detection limit, so no drug susceptibility testing was performed. Additionally, the other two cases with negative MTB-DNA results had positive IGRA results, and anti-TB therapy yielded a favorable outcome, further proving the diagnosis of ETB.

## CONCLUSION

The WHO-recommended treatment regimen for drug-susceptible TB is 2HREZ/4HR.^12^ However, the duration of anti-TB treatment varies significantly among different studies on ETB, with most studies indicating a duration between 6 and 12 months.^10^ In the present study, all patients received treatment for 12 months and responded well to anti-TB therapy.
